# Osteogenic Potential of Various Premixed Hydraulic Calcium Silicate-Based Sealers on Human Bone Marrow Stem Cells

**DOI:** 10.3390/ma18235326

**Published:** 2025-11-26

**Authors:** Na-Hyun You, Donghee Lee, Yemi Kim, Sieun Nam, Sin-Young Kim

**Affiliations:** 1 Department of Conservative Dentistry, Seoul St. Mary’s Hospital, College of Medicine, The Catholic University of Korea, Seoul 06591, Republic of Korea; ysy02320@cmcnu.or.kr (N.-H.Y.); mstldms@catholic.ac.kr (S.N.); 2 College of Medicine, The Catholic University of Korea, Seoul 06591, Republic of Korea; dong524@catholic.ac.kr; 3 Department of Conservative Dentistry, College of Medicine, Ewha Womans University, Seoul 07985, Republic of Korea; yemis@ewha.ac.kr

**Keywords:** hydraulic calcium silicate–based sealer, human bone marrow-derived stem cells, osteogenic potential

## Abstract

This study aims to compare the osteogenic potential of premixed hydraulic calcium silicate-based sealers (HCSSs) with an epoxy resin-based sealer in human bone marrow-derived stem cells (hBMSCs). Three HCSSs (White Endoseal MTA, One-Fil, EndoSequence BC Sealer) were compared with AH Plus Jet, an epoxy resin-based sealer. Disk-shaped specimens were prepared using sterilized Teflon tubes and immersed in osteogenic medium to create eluates. hBMSCs were cultured in each eluate, and osteogenic potential was assessed by alkaline phosphatase (ALP) activity (n = 6), Alizarin Red-S (ARS) staining (n = 6), quantitative real-time polymerase chain reaction (qPCR) (n = 3), and Western blot analysis. Statistical analyses were conducted using SPSS (version 24.0), with significance set at *p* < 0.05. All experimental groups exhibited higher ALP activity than the control on day 4. ARS staining of HCSSs differed significantly from AH Plus Jet on day 14 (*p* < 0.05), while White Endoseal MTA exhibited the highest intensity. qPCR revealed that EndoSequence BC Sealer induced the highest *SMAD1* expression on day 4, while One-Fil and EndoSequence BC Sealer significantly upregulated *RUNX2* expression compared with AH Plus Jet (*p* < 0.05). Western blotting confirmed that EndoSequence BC Sealer induced the highest RUNX2 protein expression. Collectively, premixed HCSSs promoted superior mineralization and *RUNX2* expression compared to conventional resin-based sealer in hBMSCs.

## 1. Introduction

For the long-term success of endodontic treatment, complete sealing of the apical region is essential [1]. Most studies have reported that confining root canal filling materials within 2 mm of the apex is ideal [2]; however, extrusion of sealers into the periodontal tissues still occurs occasionally [3]. Some studies have suggested that such extrusion may interfere with healing, leading to the development of various hydraulic calcium silicate-based sealers (HCSSs) that are biocompatible and capable of promoting healing [4].

In particular, among these, hydraulic calcium silicate-based sealers (HCSSs) have attracted attention as alternatives that can overcome the limitations of conventional sealers, owing to their biocompatibility and excellent osteogenic potential [5,6,7,8]. HCSSs not only induce a regenerative response but also provide strong antibacterial activity through their high alkalinity [9,10,11,12,13,14]. Furthermore, because they are hydrophilic compared with conventional epoxy resin-based sealers [15], and composed of finer particles [15,16], and they can penetrate into complex root canal structures and contribute effectively to achieving complete sealing [17,18,19,20,21]. Owing to these properties, HCSSs are currently being recognized in clinical practice as promising substitutes for conventional epoxy resin-based sealers [17]. With the increasing variety of HCSSs, there is a growing demand for studies evaluating their osteogenic potential in comparison with that of conventional resin-based sealers [9].

This study aimed to assess and compare the osteogenic potential of several premixed HCSSs—White Endoseal MTA (Maruchi, Wonju, Korea), One-Fil (Mediclus, Cheongju, Korea), and EndoSequence BC Sealer (Brasseler, Savannah, GA, USA)—with that of the conventional epoxy resin-based sealer AH Plus Jet (Dentsply Sirona, Konstanz, Germany) on human bone marrow-derived stem cells (hBMSCs). Furthermore, by analyzing the differences among various types of HCSSs, this study aims to provide a rationale for sealer selection.

## 2. Materials and Methods

### 2.1. Human Bone Marrow-Derived Stem Cells (hBMSCs)

This study received approval from the Institutional Review Board of the Catholic University of Korea (IRB No. ZC20TISI0736). Passage 4 hBMSCs were used in this experiment. The cell line was purchased at passage 2 from the Catholic Institute of Cell Therapy (CIC, Seoul, Korea) (BM022SS10-P2, 1.0 × 10^6^/vial) and cultured up to passage 4 prior to experimentation.

The cells were cultured in growth medium (GM) composed of α-minimum essential medium (α-MEM; HyClone, GE Healthcare Life Sciences, Pittsburgh, PA, USA), supplemented with 10% fetal bovine serum (HyClone, GE Healthcare Life Sciences), penicillin (100 U/mL) and streptomycin (100 μg/mL). The osteogenic medium (OM) used for osteogenic differentiation was prepared by supplementing α-MEM with 50 μg/mL ascorbic acid (Sigma-Aldrich, St. Louis, MO, USA), 0.1 μM dexamethasone (Sigma-Aldrich), and 10 mM β-glycerophosphate (Sigma-Aldrich). Cell maintenance was carried out in a controlled incubator environment (37 °C, 5% CO_2_, humidified), and all experiments were performed under sterile conditions.

### 2.2. Experimental Eluates of Conventional Sealer and Premixed HCSSs

In this study, the conventional sealer AH Plus Jet (Dentsply Sirona, Konstanz, Germany) and the premixed HCSSs —White Endoseal MTA (Maruchi), One-Fil (Mediclus), and EndoSequence BC Sealer (Brasseler)—were used. The composition of each material is presented in Table 1. Each sealer was handled aseptically and prepared in compliance with the manufacturer’s guidelines. Cylindrical disc-shaped specimens (6 mm in diameter, 3 mm in height) were fabricated using sterilized Teflon Tubes. Each experimental sealer was placed inside a Teflon tube, and the upper and lower surfaces of the specimens were covered with wet gauze and allowed to set in an incubator at room temperature for 72 h. They were then sterilized by ultraviolet irradiation for 4 h under clean bench conditions at room temperature.

The prepared experimental discs were immersed in OM for 1 week to release their components, and this process yielded eluates at a concentration of 5 mg/mL. The supernatant was filtered using a 0.20 μm filter (Minisart; Sartorius Stedim Biotech, Goettingen, Germany).

### 2.3. Experimental Group Classification

The sealants’ osteogenic potential was evaluated by alkaline phosphatase (ALP) assay, Alizarin Red S (ARS) staining, quantitative real-time polymerase chain reaction (qRT-PCR), and Western blot analysis.

The analyzed groups in the experiment were classified according to the type of HCSSs as follows:

Group 1. Control group: Cells cultured in medium only (no eluate) served as the control.

Group 2. AH Plus Jet group: Cells cultured with AH Plus Jet eluate

Group 3. White Endoseal MTA group: Cells cultured with White Endoseal MTA eluate

Group 4. One-Fil group: Cells cultured with One-Fil eluate

Group 5. EndoSequence BC Sealer group: Cells cultured with EndoSequence BC Sealer eluate

### 2.4. Alkaline Phosphatase (ALP) Assay

To examine the osteogenic differentiation ability of hBMSCs, ALP activity was analyzed on days 2 and 4. For cell culture, 0.7 × 10^4^ cells were placed in each well of a 24-well plate (SPL Life Sciences, Pocheon, Korea) and cultured in OM. In the experimental groups, cells were cultured with eluates prepared from each sealer. On days 2 and 4, some cells were washed twice with PBS, exposed to 20 μL of lysis buffer supplemented with 0.2% Triton X-100 (AnaSpec, Fremont, CA, USA), and subsequently incubated for 15 min at 37 °C. The adherent cells were scraped, transferred into 1.5 mL microtubes, agitated, and kept at 4 °C for 10 min. The samples were centrifuged at 2500× *g* for 10 min at 4 °C, and the resulting supernatant was harvested for ALP measurement. Subsequently, 50 μL of p-nitrophenyl phosphate (pNPP) substrate solution (AnaSpec, Fremont, CA, USA) was added to each well, stirred gently for 30 s, and incubated for 30 min at 4 °C. The absorbance was then recorded at 405 nm using a microplate reader (PowerWave XS, BioTek Instruments, Winooski, VT, USA). Each group was assessed using six independent samples (n = 6).

### 2.5. Alizarin Red S (ARS) Staining Assay

To assess mineralized nodule formation in hBMSCs, ARS staining was carried out. hBMSCs were plated in 24-well culture plates (SPL Life Sciences) at a density of 0.7 × 10^4^ cells/well and cultured with eluates obtained from the experimental discs for 7 or 14 days. Cells were fixed in 4% paraformaldehyde and subsequently stained with 2% Alizarin Red S (ARS) solution (ScienCell, Carlsbad, CA, USA) for 20 min. To release the bound dye, 10% cetylpyridinium chloride (Sigma-Aldrich) was added for 15 min, after which absorbance at 560 nm was determined using a microplate reader (PowerWave XS, BioTek Instruments, Winooski, VT, USA). Each group was evaluated using six independent samples (n = 6).

### 2.6. Gene Expression Analysis by Quantitative Real-Time Polymerase Chain Reaction (qRT-PCR)

hBMSCs were seeded in 24-well plates (SPL Life Sciences, Korea) at a density of 0.7 × 10^4^ cells/well and cultured with eluates obtained from the experimental discs for 1 and 4 days. Total RNA extraction was conducted with the RNeasy Mini Kit (Qiagen, Hilden, Germany), followed by cDNA synthesis through reverse transcription using the RevertAid First Strand cDNA Synthesis Kit (Thermo Fisher Scientific, Waltham, MA, USA). The primers used for the analysis were synthesized by Cosmo Genetech (Seoul, Korea) according to the sequences listed in Table 2. The expression of the osteogenic marker genes Runt-related transcription factor 2 (*RUNX2*) and Suppressor of Mothers against Decapentaplegic 1 (*SMAD1*) was evaluated.

Total RNA was isolated with Tri Reagent (TR118; Molecular Research Center, Inc., Cincinnati, OH, USA). After measuring RNA concentration, 400 ng was employed as input for qRT-PCR. Amplification was carried out using the iTaq Universal SYBR Green One-Step Kit (Bio-Rad, Hercules, CA, USA) on a CFX96 Real-Time PCR Detection System, and data were processed with CFX Manager software (version 3.1; Bio-Rad). Thermal cycling for qRT-PCR began with reverse transcription at 50 °C for 10 min, followed by an initial denaturation at 95 °C for 1 min. Amplification was then carried out for 45 cycles, each consisting of denaturation at 95 °C for 10 s and combined annealing/extension at 60 °C for 30 s. To obtain melting profiles, an additional 60 cycles were run at 60 °C for 5 s, with the temperature raised by 0.5 °C at each step. Relative gene expression was normalized to GAPDH as a housekeeping control and expressed as fold change compared with the control group. Each group was analyzed using three independent samples (n = 3).

### 2.7. Gene Expression Analysis by Western Blotting

After hBMSCs were cultured in the experimental eluates of each group for 3 days, cell lysis was performed with RIPA buffer (25 mM Tris–HCl pH 7.6, 150 mM NaCl, 1% NP-40, 1% sodium deoxycholate, 0.1% SDS, 1 mM PMSF, and protease inhibitors including aprotinin [1 μg/μL] and leupeptin [1 μg/μL]). The lysates were incubated on ice for 20–30 min and then centrifuged at 15,000× *g* for 10 min at 4 °C. The supernatants were collected, and protein levels were determined with a Pierce BCA assay kit (Thermo Fisher Scientific). Equal quantities of protein (15 μg) were separated by SDS–PAGE and transferred for immunoblotting with anti-RUNX2 (ab23981, 1:1000, Abcam, Cambridge, UK) and anti-GAPDH (ab9485, 1:5000, Abcam) primary antibodies. Horseradish peroxidase (HRP)-conjugated anti-rabbit IgG (1:5000, Abcam) was used as the secondary antibody. Immunoreactive bands were visualized using Immobilon Western (Merck Millipore, Burlington, MA, USA) and detected by exposing the membranes to the PXi4 Gel Documentation System (Syngene, Cambridge, UK).

### 2.8. Statistical Analysis

Statistical analyses were performed using the SPSS software program (version 24.0; IBM Corp., Armonk, NY, USA). The Shapiro–Wilk test was applied to assess the normality of data distribution. When normality was confirmed, one-way analysis of variance (one-way ANOVA) was used to evaluate differences among groups, followed by Tukey’s post hoc test for multiple comparisons. Within-group comparisons according to experimental time points were performed using the independent *t*-test. A *p*-value of <0.05 was considered statistically significant.

## 3. Results

### 3.1. ALP Activity Assay

**Figure 1:**
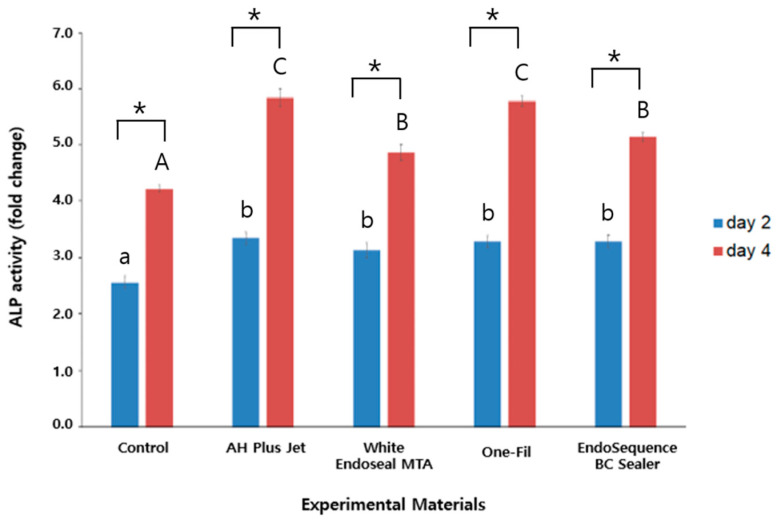
Comparison of alkaline phosphatase activity among the experimental groups on days 2 and 4. Different lowercase letters indicate statistically significant differences among experimental groups on day 2. Different uppercase letters indicate statistically significant differences among experimental groups on day 4. Asterisks (*) denote a statistical significance.

The ALP activities measured on days 2 and 4 are presented in Figure 1. In all groups, ALP levels on day 4 were markedly and significantly greater than those on day 2 (*p* < 0.05). On day 2, the control group exhibited the lowest ALP activity, whereas all experimental groups—including AH Plus Jet, White Endoseal MTA, One-Fil, and EndoSequence BC Sealer—had significantly higher activity than the control (*p* < 0.05). There were no statistically significant variations detected among the groups tested (*p* > 0.05). In contrast, clearer differences were observed on day 4: AH Plus Jet and One-Fil demonstrated the highest ALP activity, while White Endoseal MTA and EndoSequence BC Sealer also showed significantly increased activity (*p* < 0.05). The control group consistently exhibited the lowest activity.

### 3.2. ARS Staining Assay

**Figure 2:**
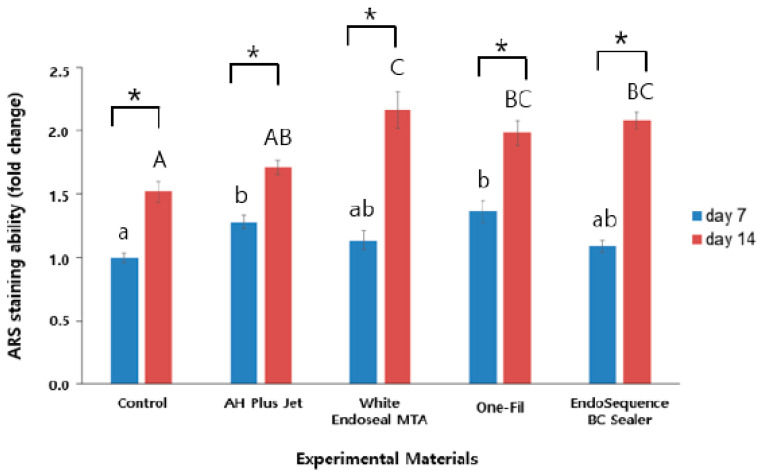
Comparison of Alizarin Red S staining values among the experimental groups on days 7 and 14. Different lowercase letters indicate statistically significant differences among experimental groups on day 7. Different uppercase letters indicate statistically significant differences among experimental groups on day 14. Asterisks (*) denote a statistical significance.

**Figure 3:**
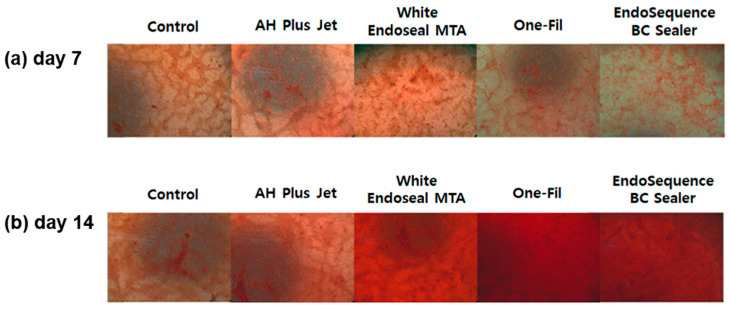
(**a**) Representative image of Alizarin Red S staining on day 7; (**b**) representative image of Alizarin Red S staining on day 14.

ARS staining was performed to evaluate the mineralization ability of each group, and the results are presented in Figure 2. The analysis was conducted on days 7 and 14, with ARS staining intensity serving as an indirect indicator of mineralized nodule formation. In all groups, a statistically significant elevation in ARS staining intensity was observed at day 14 relative to day 7 (*p* < 0.05). On day 7, the control group exhibited the lowest ARS staining intensity, whereas the AH Plus Jet and One-Fil groups showed significantly greater mineralization than did the control (*p* < 0.05). A slight elevation was detected in the White Endoseal MTA and EndoSequence BC Sealer groups relative to the control, but the difference proved not to be statistically meaningful (*p* > 0.05). On day 14, the differences among groups became more pronounced. The White Endoseal MTA group demonstrated the highest ARS staining intensity, followed by the EndoSequence BC Sealer and One-Fil groups. All three groups exhibited significantly greater mineralization than that of both the AH Plus Jet and the control groups (*p* < 0.05). Representative ARS staining images for each group are shown in Figure 3, indicating an increase in red-stained area from day 7 to day 14.

### 3.3. qRT-PCR Analysis

**Figure 4:**
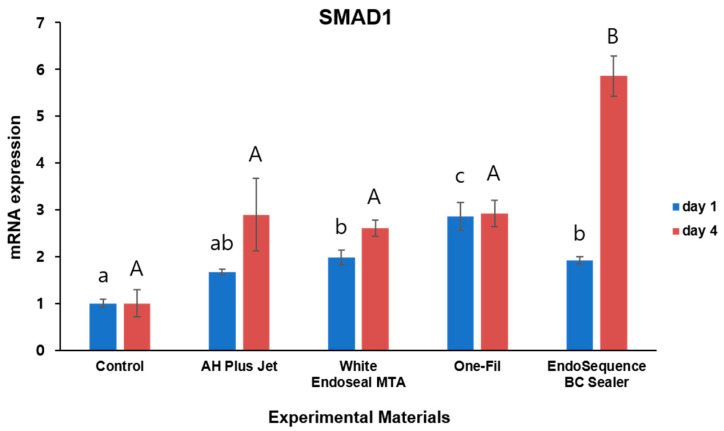
Comparison of *SMAD1* expression by qRT- PCR on days 1 and 4. Different lowercase letters indicate statistically significant differences among experimental groups on day 1. Different uppercase letters indicate statistically significant differences among experimental groups on day 4.

**Figure 5:**
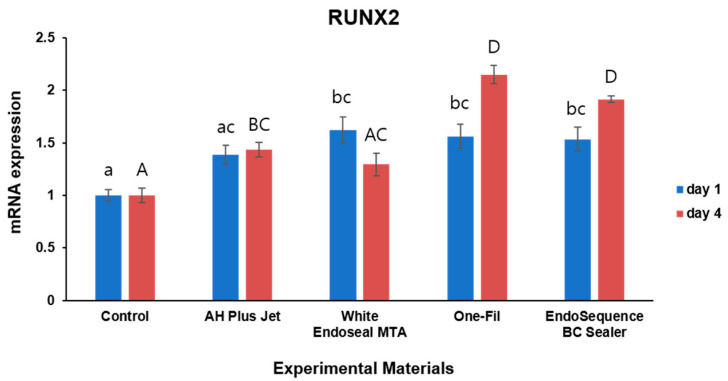
Comparison of *RUNX2* expression by qRT- PCR on days 1 and 4. Different lowercase letters indicate statistically significant differences among experimental groups on day 1. Different uppercase letters indicate statistically significant differences among experimental groups on day 4.

To investigate the expression levels of *SMAD1* and *RUNX2,* which are known osteogenic differentiation markers, qRT-PCR assays were conducted. The results are presented in Figure 4 and Figure 5.

For *SMAD1* expression, differences between the experimental groups and the control were observed at both day 1 and day 4 (Figure 4). On day 1, the One-Fil group showed the highest *SMAD1* expression, and in the White Endoseal MTA and EndoSequence BC Sealer groups, expression levels were markedly greater than those of the control, with significance confirmed (*p* < 0.05). The AH Plus Jet group displayed a trend toward increased expression, but the difference did not reach statistical significance when compared with the control (*p* > 0.05). The control group consistently showed the lowest expression levels. On day 4, the EndoSequence BC Sealer group demonstrated significantly higher *SMAD1* expression than all other groups (*p* < 0.05), whereas the remaining experimental groups did not differ significantly from the control (*p* > 0.05).

For *RUNX2* expression, increased expression was also observed in the experimental groups on both day 1 and day 4 (Figure 5). On day 1, the White Endoseal MTA, One-Fil, and EndoSequence BC Sealer groups exhibited *RUNX2* expression significantly higher than that of the control (*p* < 0.05). While expression levels in the AH Plus Jet group appeared somewhat higher than those in the control group, the change failed to achieve statistical significance (*p* > 0.05). On day 4, the One-Fil and EndoSequence BC Sealer groups demonstrated the highest *RUNX2* expression, while the AH Plus Jet group also exhibited significantly expression higher than that of the control (*p* < 0.05). In contrast, the White Endoseal MTA group showed an increase, but this did not attain statistical significance when compared with the control’s expression (*p* > 0.05).

### 3.4. Western Blot Analysis

**Figure 6:**
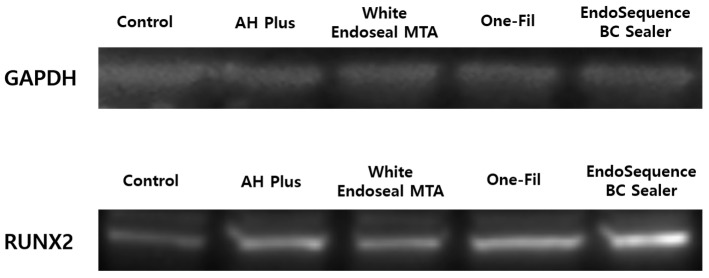
Analysis of RUNX2 protein expression according to the experimental materials by Western blot.

RUNX2 expression was investigated through Western blot, and GAPDH functioned as the control marker. Compared with the control group, all experimental groups showed increased expression of RUNX2 protein. In particular, the One-Fil and EndoSequence BC Sealer groups exhibited the strongest band intensities, indicating a marked upregulation of RUNX2 expression. The AH Plus Jet and White Endoseal MTA groups also showed a clear increase compared with the control, but to a relatively lower degree than the One-Fil and EndoSequence BC Sealer groups (Figure 6).

**Figure 7:**
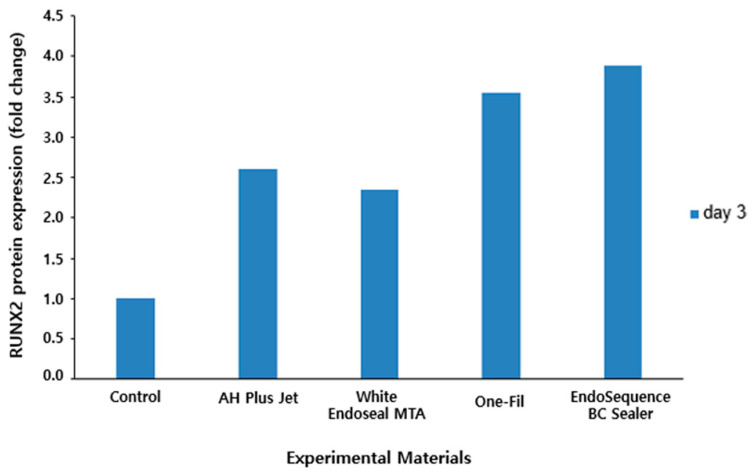
Quantitative analysis of RUNX2 protein expression by Western blot.

Following Western blot analysis of RUNX2 protein expression, band intensities were quantified and compared (Figure 7). The results revealed that all experimental groups exhibited a marked increase in RUNX2 protein expression compared with the control group. Notably, the EndoSequence BC Sealer and One-Fil groups showed the highest expression levels, with approximately 3.9- and 3.5-fold increases, respectively, which were more pronounced than those of the AH Plus Jet (approximately 2.6-fold) and White Endoseal MTA (approximately 2.3-fold) groups.

## 4. Discussion

Traditional epoxy resin-based sealers have long been regarded as the standard in terms of their physical sealing ability; however, concerns have consistently been raised regarding their initial toxicity and resulting adverse tissue responses [3]. In particular, when sealers diffuse into periodontal tissues, they may trigger inflammatory reactions or delay healing, underscoring the need for more biocompatible alternatives [3,4]. Therefore, we quantitatively compared and analyzed osteogenic potential of HCSSs, which are currently gaining attention in clinical practice for their biocompatibility and diverse applications. To provide fundamental data that may facilitate more scientific and evidence-based decision-making in sealer selection, we aimed to compare the traditional epoxy resin-based sealer AH Plus Jet with various premixed HCSSs in order to evaluate their osteogenic potential on hBMSCs. The comprehensive analysis included ALP activity, mineralized nodule formation assessed by ARS staining, and the expression of osteogenic differentiation–related genes by qRT-PCR and their proteins by Western blot.

Alkaline phosphatase (ALP) is an enzyme active during the early stages of osteoblastic differentiation and is widely used marker of the initiation of mineralized tissue formation [22]. As bone formation becomes more active, ALP expression increases, suggesting that osteoblasts are differentiating [23,24,25,26]. In this experiment, the cells treated with eluates of HCSSs maintained relatively high ALP activity throughout the experimental period, and all experimental groups exhibited a significant increase in ALP activity over time (Figure 1, *p* < 0.05). These findings suggest that HCSSs promote osteogenic differentiation, consistent with previous studies [4,27,28]. In addition, previous studies have reported that HCSSs create a highly alkaline environment that neutralizes acids secreted by osteoclasts, thereby inhibiting further degradation of mineralized tissues [29], and that they simultaneously activate alkaline phosphatase to promote the mineralization process, in line with and supporting the results of the present study [30].

We used ARS staining to quantitatively evaluate the degree of extracellular matrix mineralization in order to compare the mineralized tissue–forming capacity among the experimental groups. ARS staining is widely employed as an indicator of osteoinductive capacity ability and hard tissue formation, and, as it reflects the later stages of osteogenic differentiation, it provides information complementary to ALP analysis [31]. The analysis revealed that in all experimental groups, ARS staining intensity was significantly higher on day 14 than on day 7, indicating that mineralized tissue formation became more active over time (Figure 2, *p* < 0.05). At the early stage (day 7), the AH Plus Jet and One-Fil groups exhibited high staining intensity, suggesting their potential to induce the mineralization process of osteoblasts at a faster rate. This finding differs somewhat from previous reports, which noted that no mineralized nodules were observed in either the AH Plus group or the control group on day 7 in ARS evaluation [32]. This discrepancy may be attributable to differences in the culture medium. In the present study, OM was used, whereas the previous study employed general GM, and OM is known to facilitate mineralization more readily than GM [33]. In addition, the use of hBMSCs, which possess high differentiation potential and are sensitive to inductive signals, instead of mature osteoblasts as in the previous study, may have led to a more pronounced mineralization response [34]. On day 14, the pattern shifted, with White Endoseal MTA, EndoSequence BC Sealer, and One-Fil showing significantly higher activity. These HCSSs are interpreted to exert a stronger influence on osteoblastic mineral deposition and hard tissue formation at the later stage. In other experimental studies, the White Endoseal MTA and EndoSequence BC Sealer groups also demonstrated significantly greater calcium nodule formation at day 15 that did the AH Plus group [35]. According to the scoping review by Estivalet et al., among a total of 53 studies published between 2011 and 2022, ARS staining was frequently employed as a key indicator to assess the mineralization capacity of HCSSs, and most of these studies reported that HCSSs exhibited calcium deposition ability superior to that of the control group (AH Plus) [36], consistent with the findings of the present experiment.

We analyzed the expression of *SMAD1* and *RUNX2*, key transcription factors in the regulation of osteogenic differentiation [37], by qRT-PCR to evaluate the osteogenic potential of each HCSS in comparison with AH Plus Jet. *SMAD1* is an upstream signaling factor that induces osteoblast differentiation through the BMP-Smad pathway, whereas *RUNX2* is known as a master regulator that controls osteoblastic differentiation and maturation [24,38]. Therefore, the expression patterns of these genes can serve as biological indicators reflecting the effects of HCSSs on osteoblastic differentiation, as in previous studies that examined *RUNX2* expression by qRT-PCR [27,35,39,40].

Throughout the entire experimental period, differences in *SMAD1* gene expression were observed between the experimental and control groups. On day 1, cells treated with all HCSSs except AH Plus jet exhibited a significant increase (Figure 4, *p* < 0.05), suggesting that these sealers positively influenced the early activation of signaling pathways. On day 4, cells treated with EndoSequence BC Sealer showed the highest expression among all groups. In previous studies, rat pre-osteoblasts exposed to EndoSequence BC Sealer showed increased expression of *BMP1*, *BMP2*, and *ALP* genes. The significant upregulation of *BMP2*, an upstream regulator of the BMP-Smad pathway [41], supports the possibility that this pathway remained continuously activated over time.

In this study, all HCSSs induced a significant increase in *RUNX2* expression compared with that in the control group at the early time point (day 1) (Figure 5, *p* < 0.05). This contrasts with some previous studies that failed to demonstrate the early osteogenic induction capacity of HCSSs [40]. This discrepancy, similar to the trend observed between the present and previous studies in the ARS results, may be attributable to differences in the culture conditions and cell types used [40]. In terms of culture conditions, the previous study used a general GM without osteogenic inducers [40], whereas the present study employed an OM supplemented with osteogenic factors. In clinical practice, HCSSs come into contact with periodontal and bone tissues, where physiological processes such as inflammatory responses, tissue regeneration, and bone formation take place [42]. Such healing processes resemble the environment created by the components of OM, namely ascorbic acid, β-glycerophosphate, and dexamethasone; therefore, the findings observed in this study can be considered to support clinical relevance. In addition, the previous study used pre-osteoblasts (MC3T3-E1) as a standard model for osteoinduction [40], and it is possible that these cells are relatively less sensitive to early osteogenic responses than are the hBMSCs used in this study. Nevertheless, hBMSCs, as human-derived cells, are of particular significance because they bear a closer relevance to clinical applications. On day 4, only the White Endoseal MTA group showed a non-significant increase in *RUNX2* expression (Figure 5, *p* > 0.05), which may be attributable to factors such as its ion release rate, initial pH, and compositional differences [4,10,43,44]. While EndoSequence BC Sealer and One-Fil do not contain dimethyl sulfoxide (DMSO), White Endoseal MTA contains approximately 10–30% DMSO. DMSO has been reported in previous studies to exert various biological effects, including cytotoxicity, regulation of gene expression at the transcriptional level, and inhibition of differentiation through suppression of glycosaminoglycan synthesis [45,46,47], and these properties may have negatively influenced the cellular responsiveness and osteogenic gene expression of the sensitive hBMSCs. Its relatively small portion of tricalcium silicate at 5–15% compared with those of other HCSSs might also have affected the lower *RUNX2* expression results in the White Endoseal MTA group [6,10,48]. EndoSequence BC Sealer and One-Fil have higher proportions of calcium silicate; therefore, they exhibited not only a rapid initial response but also sustained *RUNX2* expression (Figure 5, *p* < 0.05) [49,50,51].

We used Western blot to examine the expression level of RUNX2, a key transcription factor related to osteogenic differentiation. The expression level of RUNX2 is regarded as a major indicator for indirectly assessing the osteogenic potential of materials [38,52,53]. Quantitative analysis of protein expression by Western blot revealed an increasing trend of RUNX2 expression in all the experimental groups compared with the control group, suggesting that they possess clinically favorable properties in terms of bone regeneration capacity (Figure 6 and Figure 7). This finding is consistent with previous studies that investigated RUNX2 expression of HCSSs using Western blot analysis [27,54]. In particular, the One-Fil and EndoSequence BC Sealer groups in this study exhibited a more pronounced increase in expression than did AH Plus Jet, demonstrating that these two HCSSs are better inducers of osteoblastic differentiation. These results are consistent with the earlier qPCR analysis of *RUNX2* gene expression, and the protein-level findings likewise support the osteogenic potential of HCSSs.

In this study, the osteogenic potential of a traditional epoxy resin-based sealer (AH Plus Jet) and three HCSSs (White Endoseal MTA, One-Fil, and EndoSequence BC Sealer) was comprehensively compared and evaluated through analyses of ALP activity, ARS staining, and the expression of osteogenic differentiation–related genes and proteins. The cells treated with the HCSSs generally maintained high ALP activity and demonstrated superior late-stage mineralization potential as confirmed by ARS staining. In addition, a significant increase in *SMAD1* and *RUNX2* expression indicated their positive influence on osteoblastic differentiation. In particular, One-Fil and EndoSequence BC Sealer exhibited high reproducibility and strong osteogenic potential throughout the experiments, suggesting that HCSSs may be a favorable option for promoting periapical healing in clinical practice.

However, this study was conducted in vitro using hBMSCs, and thus there are inherent limitations in fully replicating the clinical environment. In addition, biosafety, including cytotoxicity and long-term tissue responses, was not encompassed. Another limitation of this study is that it did not take into account the physical properties of HCSSs, such as mechanical strength, flowability, solubility, and thermal resistance. Since these properties must be ideally maintained within the root canal even after obturation to ensure the long-term success of endodontic treatment, further evaluation in this regard is warranted. At last, we performed the experiments after complete setting of each experimental disc, and AH Plus Jet showed somewhat favorable osteogenic potential. Therefore, it is necessary to evaluate AH Plus Jet in its freshly mixed state.

Nevertheless, this study provided scientific evidence of the biological responses of cells treated with various HCSSs by comprehensively comparing and analyzing their osteogenic potential. Premixed HCSSs promoted superior mineralization capacity and higher *RUNX2* gene expression compared to epoxy resin-based sealer in hBMSCs. The results will be a useful reference for the selection of root canal filling materials and serve as foundational data for future clinical applications.

## Figures and Tables

**Table 1:** The manufacturer and chemical constituents of each material tested in this study.

Material	Manufacturer	Composition	Batch Number
AH Plus Jet	Dentsply DeTrey GmbH, Konstanz, Germany	Paste A Bisphenol–A epoxy resin Bisphenol–F epoxy resin Calcium tungstate, Zirconium oxide Silica, Iron oxide pigments Paste B Dibenzyldiamine, Aminoadamantane Tricyclodecane–diamine Calcium tungstate, Zirconium oxide Silica, Silicone oil	2211000712
White Endoseal MTA	Maruchi, Wonju, Korea	Zirconium dioxide 50–75% Dimethyl sulfoxide 10–30% Tricalcium silicate 5–15% Lithium carbonate <0.5% Thickening agent <6%	KD220713
One-Fil	Mediclus, Cheongju, Korea	Calcium silicate compound 35–40% Zirconium dioxide 40–45% Thickening Agent <11.5%	OS35T621
EndoSequence BC Sealer	Brasseler, Savannah, GA, United States	Zirconium oxide Calcium silicates Calcium phosphate monobasic Calcium hydroxide Filler Thickening agents	18004SP

**Table 2:** Primer sequences applied for qRT-PCR.

Runt-Related Transcription Factor 2 (*RUNX 2*)	Forward: 5′-AAG TGC GGT GCA AAC TTT CT-3′ Reverse: 5′-TCT CGG TGG CTG CTA GTG A-3
Suppressor of Mothers against Decapentaplegic (*SMAD1*)	Forward: 5′-CCA CTG GAA TGC TGT GAG TTT CC-3′ Reverse: 5′-GTA AGC TCA TAG ACT GTC TCA AAT CC-3′
GAPDH	Forward 5′-TGT CAT CAA CGG GAA GCC-3′ Reverse 5′-TTG TCA TGG ATG ACC TTG-3′

## Data Availability

The datasets used/or analyzed during the current study are available from the corresponding author on reasonable request.
